# Supplementary material for: Unraveling the Molecular Tumor-Promoting Regulation of Cofilin-1 in Pancreatic Cancer
Source: Cancers (Basel). 2021 Feb 10;13(4):725. doi: 10.3390/cancers13040725 (PMC7916621; doi:10.3390/cancers13040725)
Supplement: Supplementary file 1 [file cancers-13-00725-s001.pdf]

Supplementary Materials: Unraveling the Molecular Tumor-Promoting Regulation of Cofilin-1 in Pancreatic Cancer

Silke D. Werle, Julian D. Schwab, Marina Tatura, Sandra Kirchhoff, Robin Szekely, Ramona Diels, Nensi Ikonomi, Bence Sipos, Jan Sperveslage, Thomas M. Gress, Malte Buchholz and Hans A. Kestler

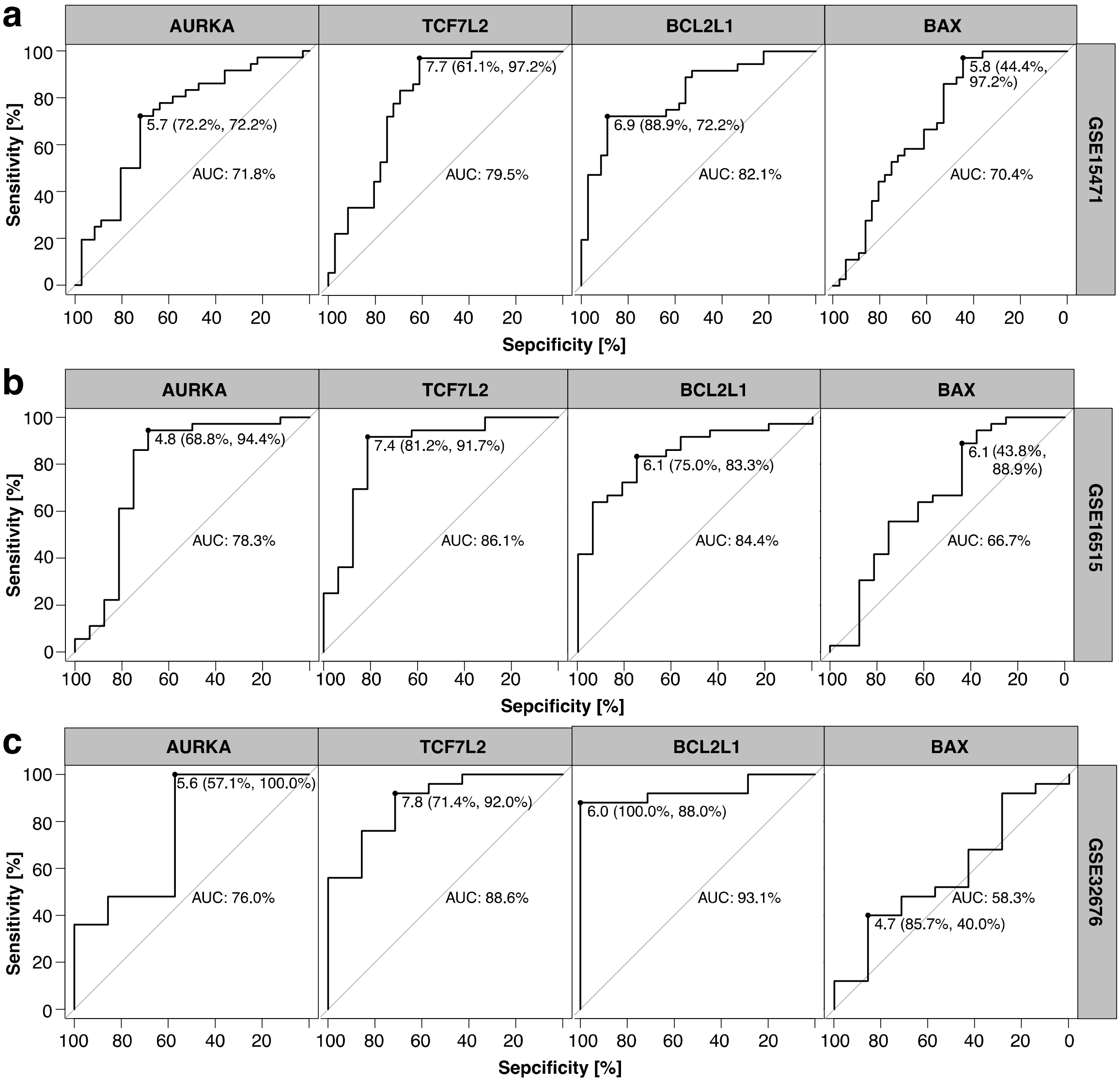

Figure S1. ROC curves to define binarization thresholds. Since all activity values within the Boolean network model are binary, the expression data from microarrays from pancreatic tissues and normal tissues was binarized using a ROC curve. The area under the curve (AUC) describes how well the classifier performs. If the AUC value would be 50%, the classifier is not better than a classification by chance. The threshold differentiating between normal and the tumor is the value where both the sensitivity and specificity of the classification are maximal. Expression values from the microarrays which are below the defined threshold are classified as 0 and values above this threshold as 1. The expression levels of the genes AURKA, TCF7L2, BCL2L1, and BAX were independently binarized for the three microarray datasets GSE15471 (**a**), GSE16515 (**b**) and GSE32676 (**c**).

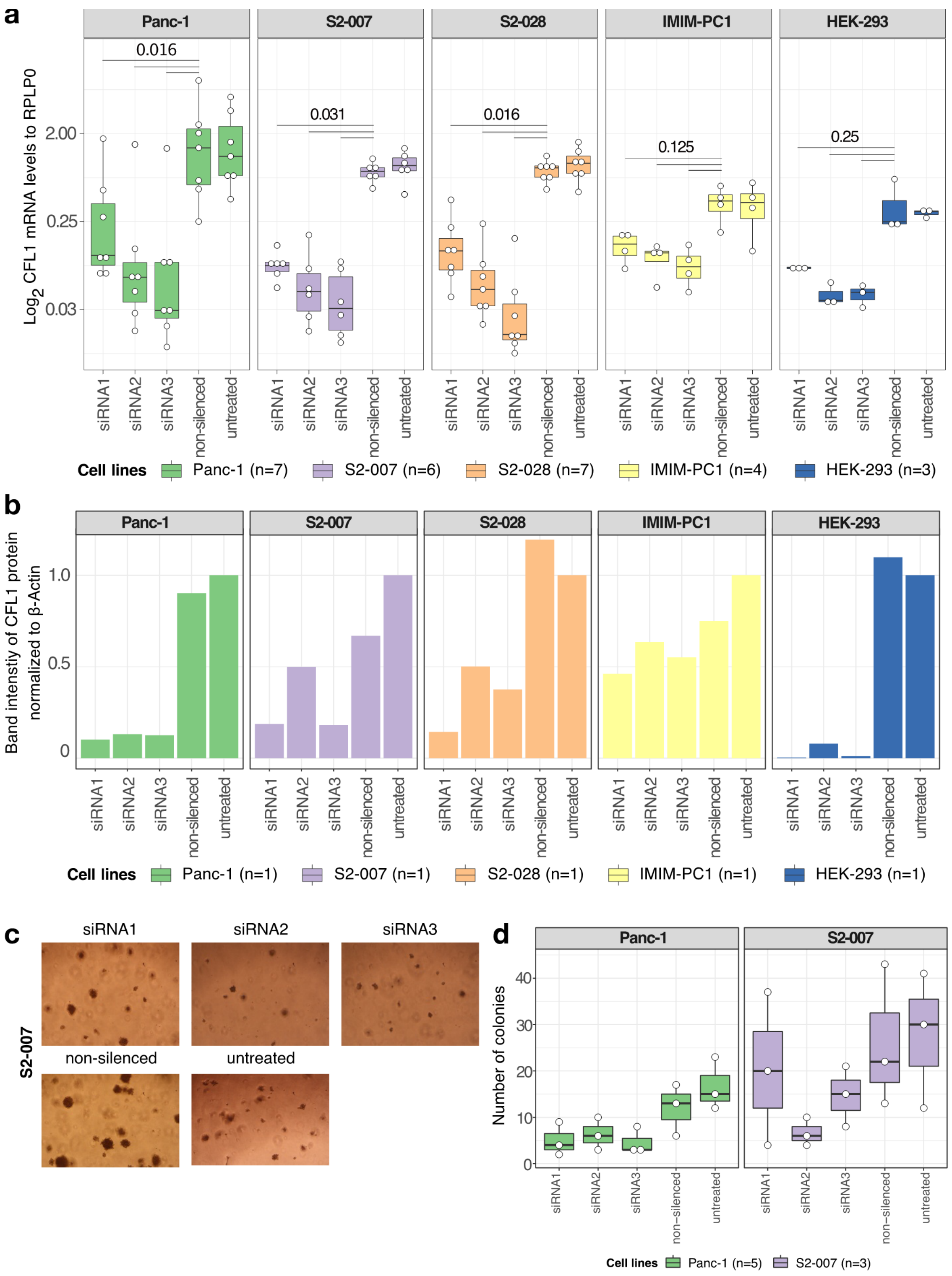

Figure S2. CFL1 knockdown. (a,b) CFL1 knockdown was performed by three independent siRNAs in four pancreatic cancer cell lines (Panc-1, S2-007, IMIM-PC1, and S2-028) and one control cell line (HEK-293). (**a**) Quantitative real-time polymerase chain reaction (qRT-PCR) demonstrates CFL1 knockdown efficiencies of 60–90% on the mRNA level. CFL1 expression values are depicted relative to the housekeeping gene RPLP0. Boxplots depict the median with the first and third quartiles. The number of samples (n) represents biological replicates. Statistical analyses were performed using the Wilcoxon test and p-values ≤ 0.05 were considered as significant. (**b**) Quantification of CFL1 protein levels normalized to β-actin loading control from the western blot of figure 1b. (**c**) Following transient silencing of CFL1 expression, a reduced colony size of S2-007 cells growing in soft agar was observed. Micrographs in 40x magnification are representatives of the evaluation (**d**). Panc-1 as well as S2-007 cells showed a reduced number cell colonies following transient CFL1 silencing in comparison to cells treated with non-silencing control siRNA.

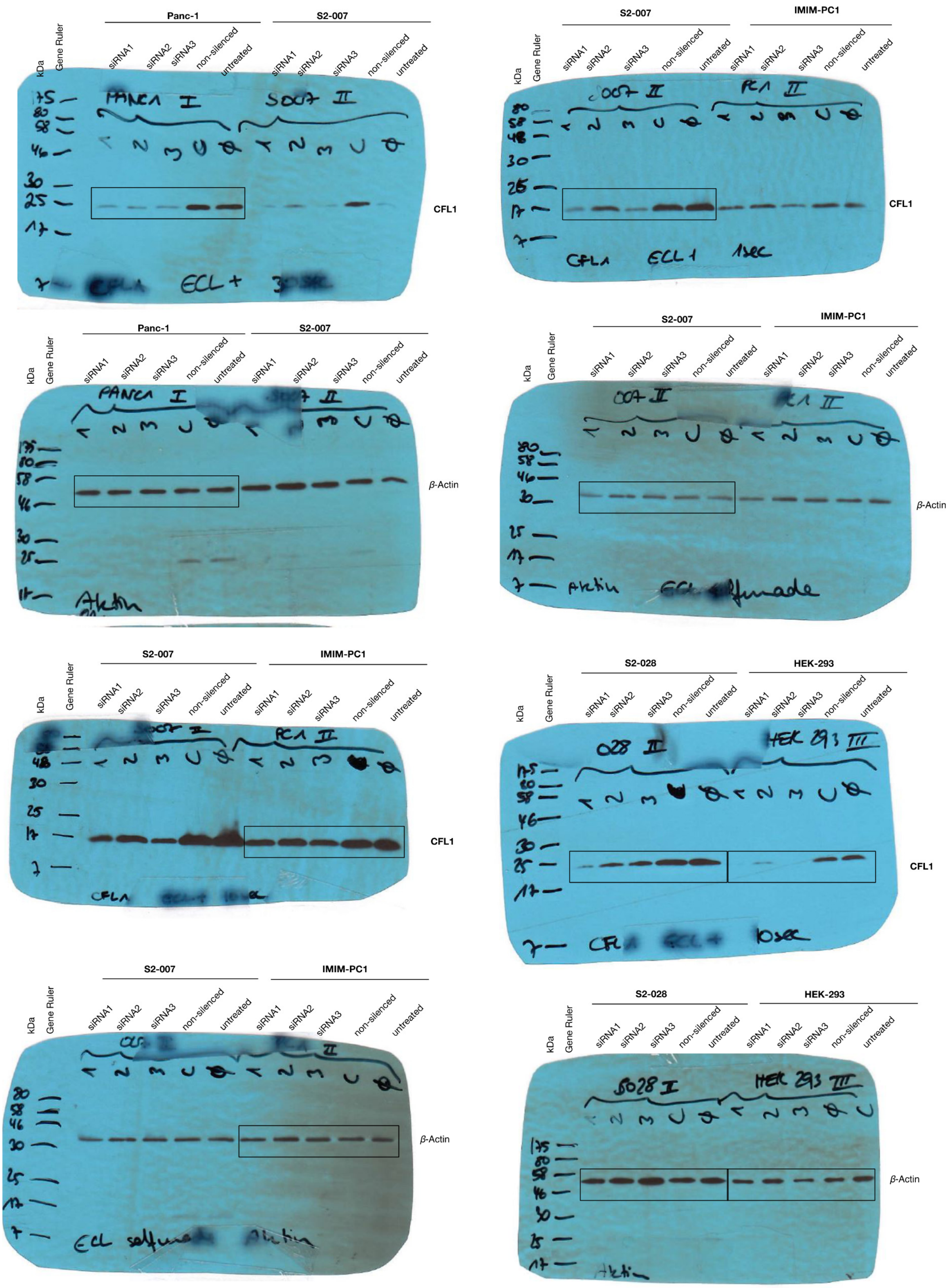

Figure S3. Uncropped Western blot images of CFL1 expression in various pancreatic cancer cells. CFL1 knockdown was performed by three independent siRNAs in four pancreatic cancer cell lines (Panc-1, S2-007, IMIM-PC1, and S2-028) and one control cell line (HEK-293). CFL1 knockdown was confirmed on the protein level. β-actin was used as an equal loading control. Depicted are the uncropped membranes from figure 1b in the main.

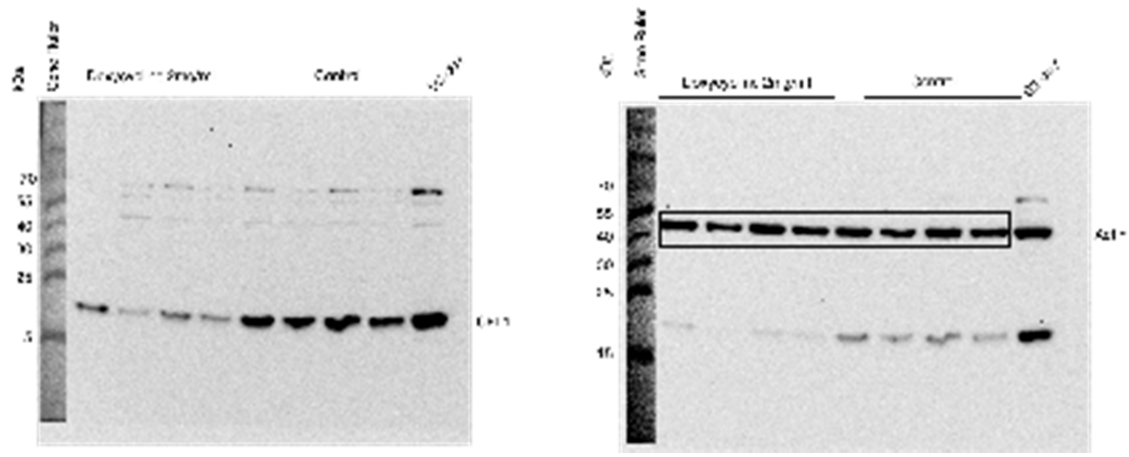

Figure S4. Uncropped Western blot images of doxycycline induced CFL1 expression in nude mice. S2-007 cells with doxycycline-inducible CFL1 knockdown were injected into nude mice. One-half of the mice (n = 6) were treated with doxycycline via drinking water to induce CFL1 repression. After the explantation of the tumors proteins were extracted from tissues and analyzed for the CFL1 level. Western blots confirmed repression of CFL1 in the treatment group. β-Actin served as a loading control. Depicted are the uncropped membranes from figure 1e in the main.

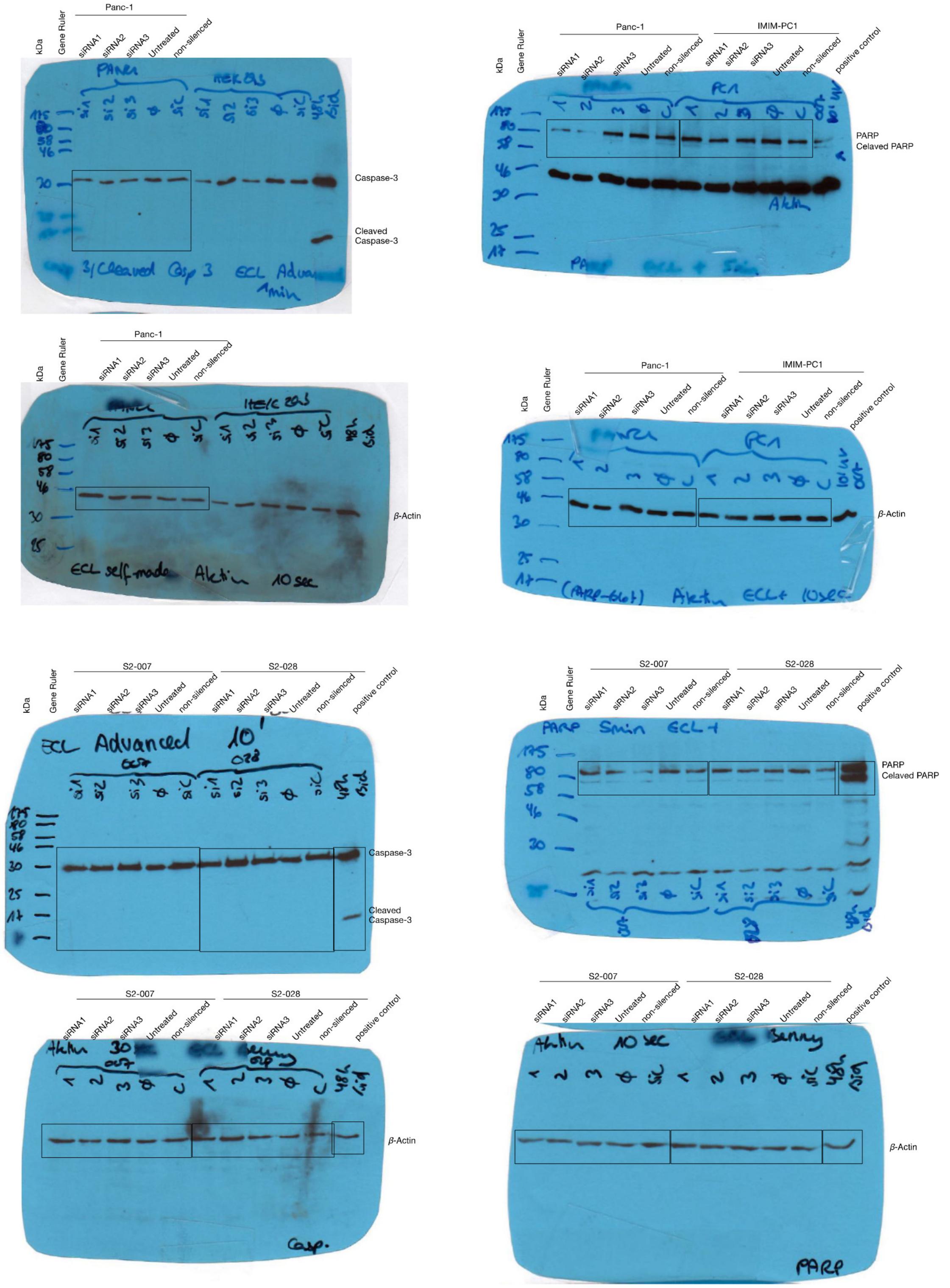

Figure S5. Uncropped Western blot images of apoptosis markers. Western blot analyses with antibodies against full-length and cleaved caspase 3 as well as full length and cleaved poly (ADP-ribose) polymerase (PARP) were performed to check for apoptosis-inducing effects of CFL1 knockdown. UV-treated S2-007 cells served as positive controls for both markers. β-actin was used to ensure equal loading. No cleavage of caspase-3 or PARP was observed following CFL1 knockdown. The results are representatives of three independent experiments. Depicted are the uncropped membranes from figure 2a in the main.

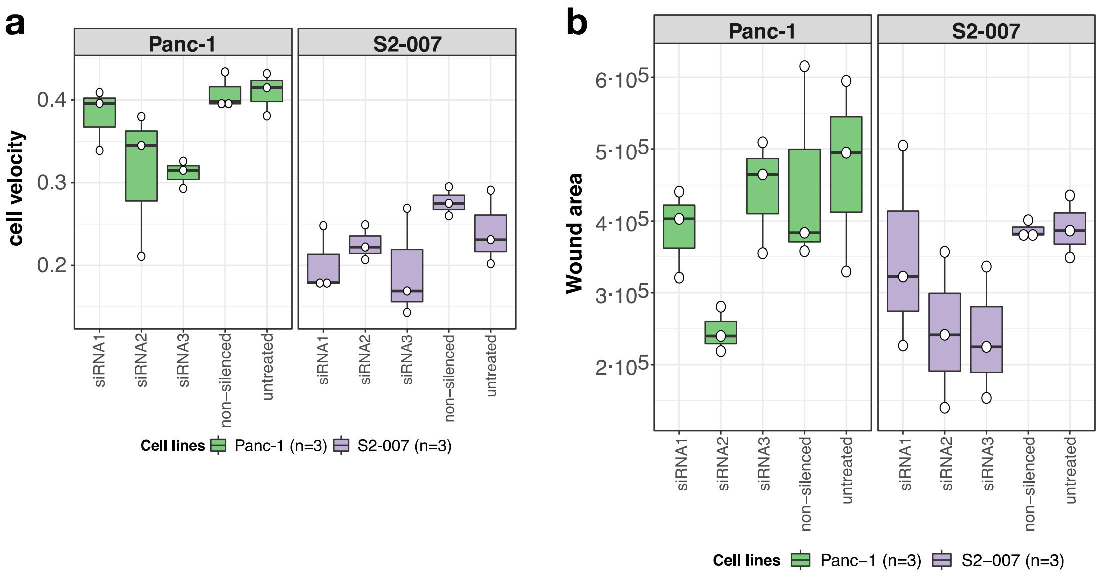

Figure S6. CFL1 silencing inhibits migration. (**a**) Automated time-lapse microscopy was used to determine average cell velocities of individual cells in subconfluent cultures or (**b**) rates of wound closure in confluent cultures of Panc-1 and S2-007 cells. The boxplots depict the median with the first and third quartiles.

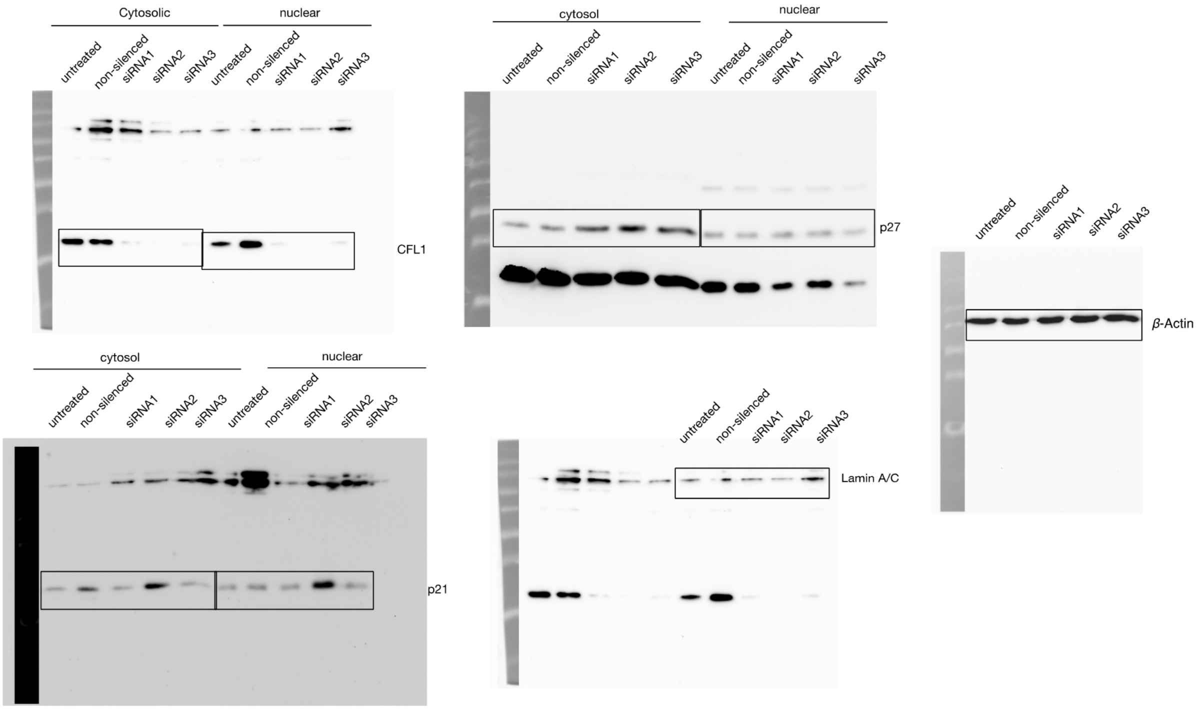

Figure S7. Uncropped Western blot images of cytosolic and nuclear protein distribution. Western blot analyses with antibodies against CFL1 as well as against cyclin-dependent kinase inhibitors 1A and 1B (p21, p27) were performed to check for involvement in G1 cell cycle arrest. β-actin and Lamin A/C were used to ensure equal loading. Neither cytoplasmic nor nuclear cell fractions of S2-007 cells showed an impact of CFL1 silencing on p21 and p27 protein level thereby excluding the role of these proteins in proliferation regulation. Depicted are the uncropped membranes from figure 4c in the main.

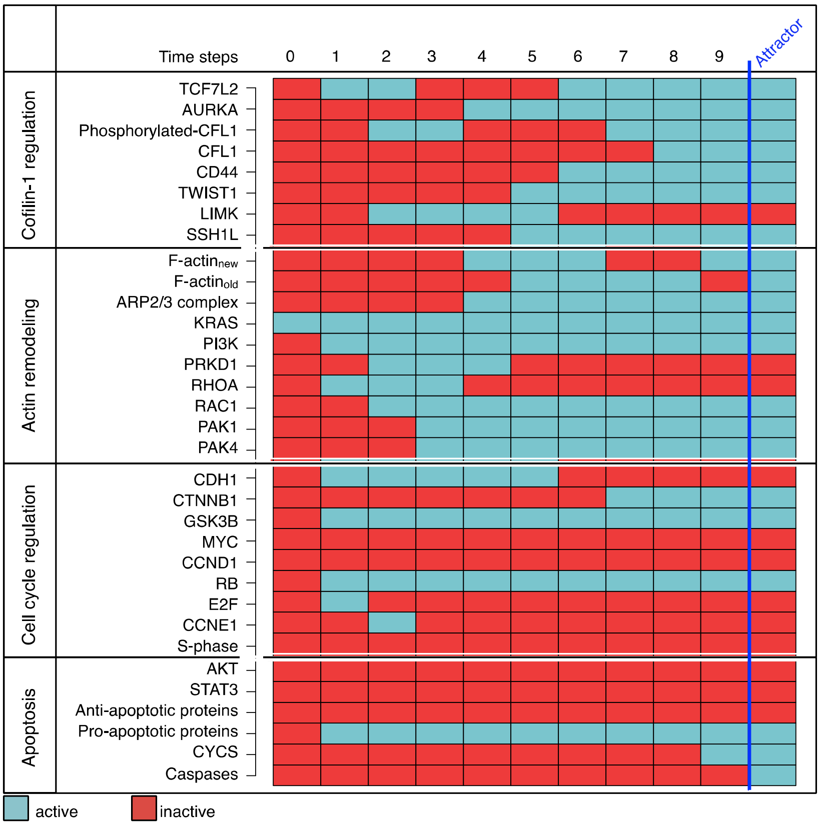

Figure S8. In-silico STAT3 knockout in pancreatic cancer cells. The simulation of a signaling cascade with an in-silico STAT3 knockout yield a single state attractor representing cell cycle arrest and induction of apoptosis. The signaling cascade starts from an initial state with only active KRAS as present in 90% of pancreatic cancer patients and proceeds in distinct time steps towards the attractors. The network components are listed on the left, while the state of each protein is represented by blue (=active) and red (=inactive) rectangles.

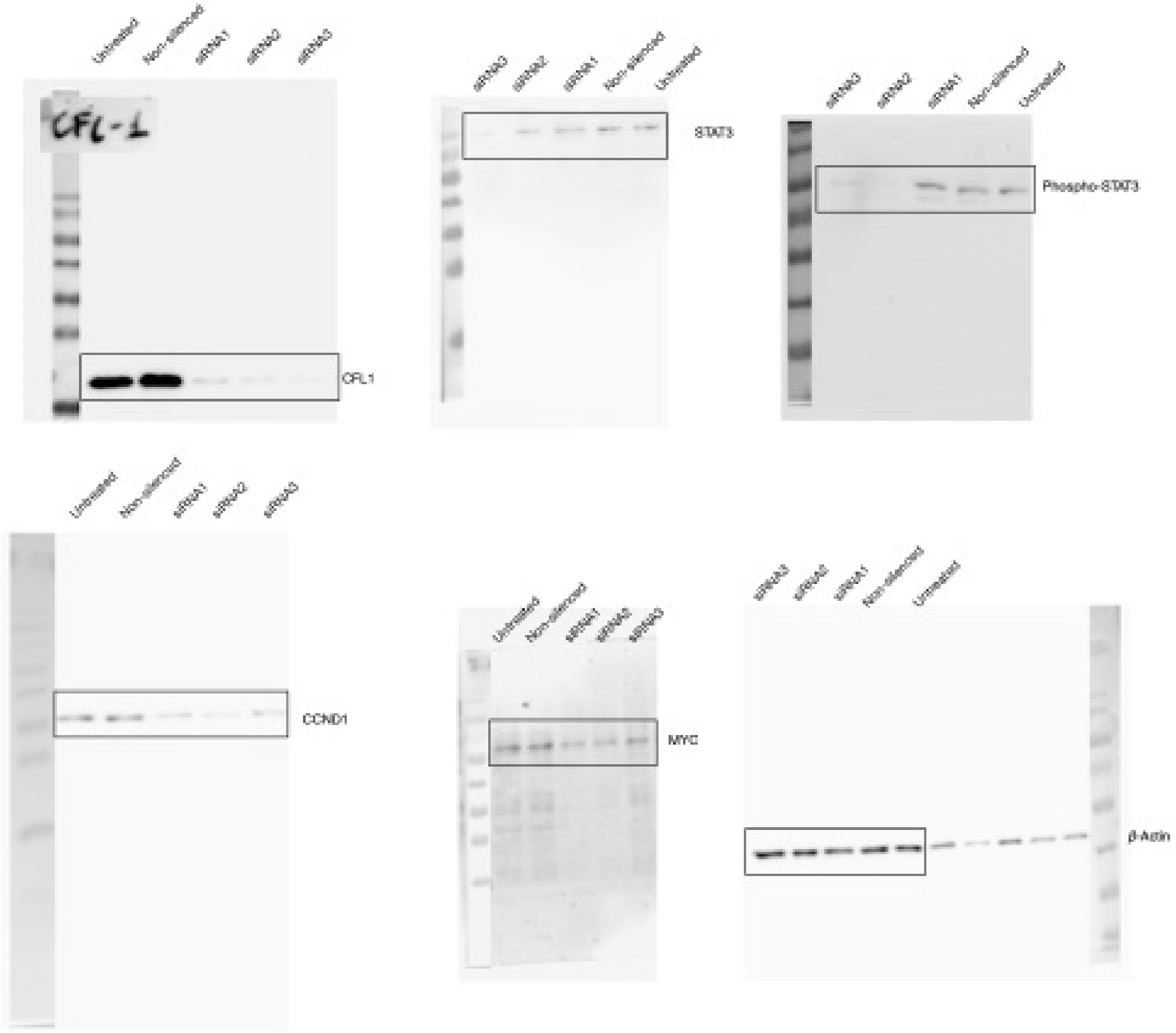

Figure S9. Uncropped Western blot images of model-suggested cell cycle regulation. Western blot analyses were performed to confirm the model suggested CFL1 regulation on cell cycle regulators in S2-007 cells. CFL1 silencing is accompanied by a reduction of total and active (phosphorylated) STAT3 as well as a decrease of CCND1 and MYC. Depicted are the uncropped membranes from figure 4d in the main.

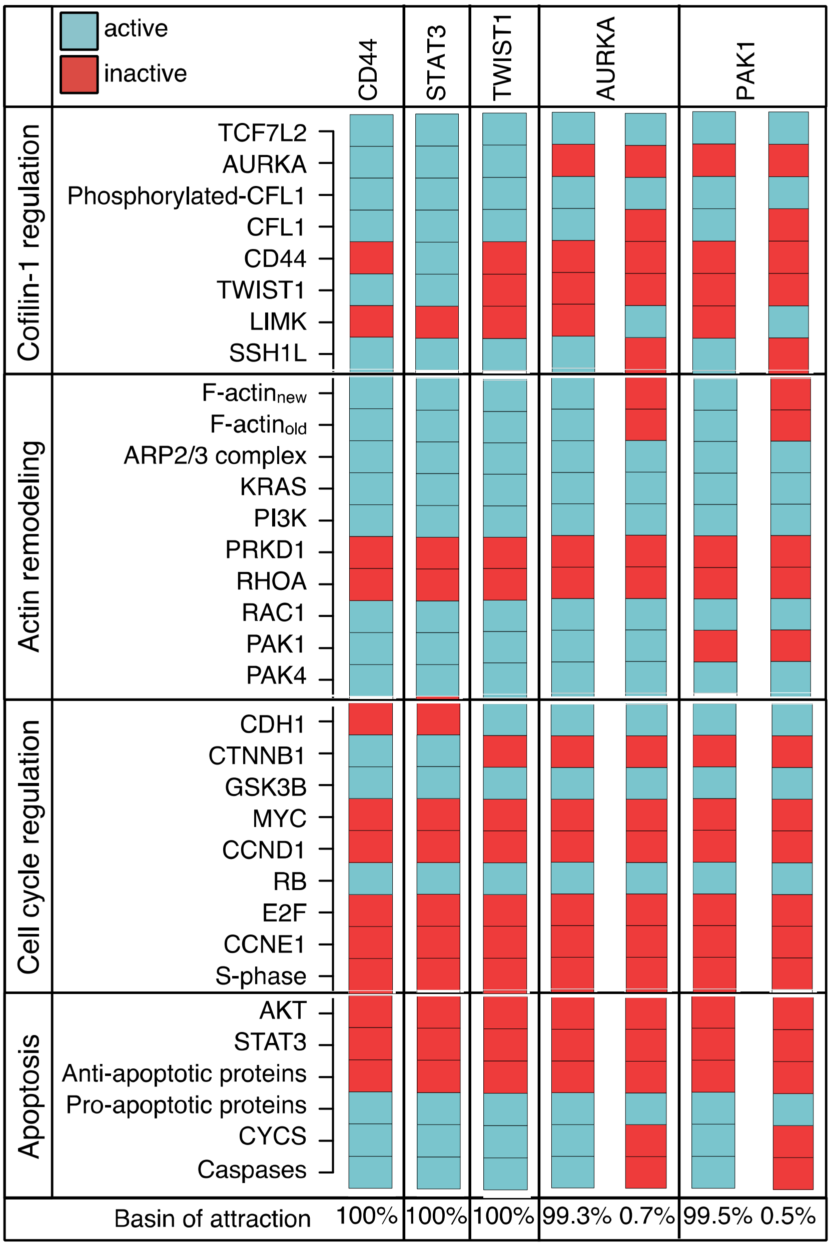

Figure S10. In-silico screening for therapeutic targets. Automated model intervention screening identified a list of proteins that might induce apoptosis. Displayed is the long-term behavior of the model after introducing the model-suggested interventions. An in-silico knockout of CD44, STAT3, or TWIST1 induced apoptosis (active caspases) in all model simulations. The majority of simulations with inactive AURKA or PAK1 also resulted in apoptosis while a minority of initial states ended in an attractor without apoptosis but inactive proliferation or migration trades. Proteins are listed on the lift while the activity of each network component is displayed by colored rectangles.

Table S1. Model-based suggestions to induce apoptosis in pancreatic cancer cells. Suggested single interventions or combinations of interventions that induce apoptosis (=active caspases) in the CFL1 model.

Factors to test for all combinations	F-actin$_{new}$, F-actin$_{old}$, ARP2/3, KRAS, PI3K, PRKD1, RHOA, RAC1, PAK1, PAK4, LIMK, SSH1L, phosphorylated-CFL1, CFL1, AURKA, CD44, TCF7L2, TWIST1, CDH1, CTNNB1, GSK3B, MYC, CCND1, RB, E2F, CCNE1, S-phase, AKT, STAT3, anti-apoptotic proteins, pro-apoptotic proteins, CYCS
Maximum set size for combination	2
Factors of interest in attractor comparison	Caspases
Candidates for meaningful perturbations	CD44 = 0<br>STAT3 = 0<br>TWIST1 = 0<br>Phosphorylated-CFL1 = 0, CYCS = 1<br>CFL1 =1, PAK1 = 0<br>CFL1 = 1, AURKA = 0<br>F-actin$_{old}$ = 1, PAK1 = 0<br>F-actin$_{old}$ = 1, AURKA = 0<br>F-actin$_{new}$ = 1, PAK1 = 0<br>F-actin$_{new}$ = 1, AURKA = 0<br>PI3K = 0, CYCS = 1<br>AKT = 0, anti-apoptotic proteins = 0<br>AKT = 0, CYCS = 1<br>PAK1 = 0, CYCS = 1<br>AURKA = 0, CYCS = 1<br>SSH1L = 1, PAK1 = 0<br>SSH1L = 1, AURKA = 0
